# From corners to community: exploring medical students’ sense of belonging through co-creation in clinical learning

**DOI:** 10.1186/s12909-024-05413-2

**Published:** 2024-04-30

**Authors:** Valerie Isobel Rae, Samantha Eve Smith, Samantha Rae Hopkins, Victoria Ruth Tallentire

**Affiliations:** 1 Medical Education Directorate, Medical Education Centre, NHS Lothian, Western General Hospital, Crewe Road South, Edinburgh, EH4 2XU UK; 2 Scottish Centre for Simulation and Clinical Human Factors, Forth Valley Royal Hospital, Larbert, FK5 4WR UK

**Keywords:** Co-creation, Belonging, Medical education, Identity, Integration

## Abstract

**Background:**

Belonging is critical for the development and wellbeing of medical students. Belonging, particularly within a ‘relational being’ paradigm, presents a significant challenge for students, especially within clinical learning environments. Co-creation is a learning relationship in which students are actively involved in the education process. It is inherently relational and promotes belonging within higher education environments. Little is known about utilising co-creation *in* the curriculum, within medical education. The aim of this study was to explore medical students’ experience of co-creation of learning resources within the clinical learning environment.

**Methods:**

Following ethical approval, medical students were invited to become co-creators of a learning bulletin resource, within the paediatric acute receiving unit, at a paediatric teaching hospital. Interpretative phenomenological analysis (IPA) was used to enable an in-depth exploration of how medical students experienced co-creation within the clinical learning environment. Medical students participated in semi-structured interviews about their experience, which were transcribed verbatim and analysed using IPA. The analysis integrated individual lived experiences into an analytic summary.

**Results:**

Nine medical students participated. Three group experiential themes were identified: identity maturation; learning community and workplace integration. The support found within this co-created learning community, along with maturation of their identity, allowed the participants to experience a challenge to their existing worldview. This shift in perspective resulted in them responding and behaving in the workplace in new ways, which enabled them to belong as themselves in the clinical learning environment. These findings were situated within the developmental concept of self-authorship, as well as contributing to a new understanding of how co-creation promoted social integration.

**Conclusions:**

Co-creation enabled students to learn in a meaningful way. The relational power of co-creation, can be harnessed to deliver participatory learning experiences, within our increasingly complex healthcare environment, to support the learning, development and integration of doctors of the future.

**Supplementary Information:**

The online version contains supplementary material available at 10.1186/s12909-024-05413-2.

## Background

In the melee of modern clinical environments, medical students often struggle to belong [1]. The student experience is rotational and transitory, and students are frequently anonymous within clinical teams. Medical students endure more psychological distress than matched peers, which is intensified when entering the clinical learning environment [2, 3]. Belonging is a fundamental pre-requisite of human development and wellbeing [4]. Educator responsibility to nurture learning environments that promote belonging has amassed increasing attention in the post-pandemic world. Conceptions of belonging incorporate feelings of acceptance, being valued and ‘fitting in’ [5]. A recent alternative paradigm of belonging, referred to as relational being, offers a challenge to the dominant understanding of students belonging *to* and ‘fitting in’ with the university [6]. Instead, it encourages a relational view of students as, “being within an ecology” which “explicitly values what they bring”, as well as their diversity [6]. In response, attention to learning structures and processes that promote students’ being within this paradigm, is of interest, as a previously unexplored solution.

Co-creation is a close collegiate relationship between students and teachers that welcomes students’ perspectives and actively involves them in the teaching and learning process [7]. Co-creation gifts a potential solution to students’ challenges with belonging in the clinical learning environment because it is inherently relational. Within higher education environments, co-creation enables students to become valued partners, and promotes creativity and critical thinking skills [7, 8].

Through a mutualistic symbiotic learning relationship, co-creation aims to enrich teaching and learning, via a social, dialogic and creative process, resulting in something new [8, 9]. Co-creation is often situated alongside ‘students as partners’ and ‘student engagement’ strategies [10]. However, during co-created learning and teaching there is an expectation that students will share responsibility, decision-making and embrace enhanced agency within meaningful relationships with teachers, recently described as ‘agentic engagement’ [11, 12]. In relation to higher education courses that have adopted co-created learning and teaching, students describe the experiences as relationship-enhancing, grounded in trust, and immensely rewarding [8]. Meaningful learning is defined as the interaction between existing knowledge and that which is newly acquired [13]. Co-creation may introduce students to meaningful participatory learning relationships, with consequential learning benefits as they negoitate between existing and new knowledge.

Co-created learning relationships within higher education benefit students, as a result of them becoming valued partners [14–17]. Key features of co-creation include student empowerment, reciprocity in the learning relationship with teachers and flattening of hierarchy. Within this context, education becomes a shared endeavour of constructing and negotiating understanding *with*, rather than *to*, the student [8, 18, 19]. Successful co-created learning and teaching experiences are generated by ensuring reciprocal respect and distributed ownership, enabled by explicit communication relating to the intention of the experience [20]. Failure to enact these attributes has negative consequences [14]. The bidirectional relationship within co-creation is empowering for basic psychological needs, defined by self-determination theory; autonomy, belonging and competence [21]. These needs must be met to drive and promote intrinsic motivation [21]. Co-creation offers a new space for students to add their value to learning and teaching.

In addition to offering a new form of learning relationship, co-creation also enables new roles. Hence, this becomes an opportunity for transformation, and may inspire critical examination of pervasive norms [22, 23]. Re-examination is essential within our increasingly complex modern healthcare environments, and assists in attending to concerns about sustainability of education [24]. Co-creating with students promotes critical thinking and creative skills [8], which are areas that the educational research community has identified to be of key importance for future research [25]. In sum, the values of co-creation enable students to explore new ways of thinking and being, which may help to create learning environments in which students can flourish whilst navigating the complexity of the healthcare setting.

Despite copious evidence of co-creation’s benefits within higher education, its relational pedagogy (underpinned by social constructivism), has yet to be fully harnessed within medical education [26, 27].

Co-creation can take multiple forms both *in* and *of* the curriculum. Co-creation *of* the curriculum is an activity that informs educational design (e.g., curricular re-design or module re-design), usually prior to learning and teaching taking place. Whereas, co-creation *in* the curriculum is an activity that creates learning and teaching during a programme or course. For example, students and teachers jointly determining a title and topic for an essay or creating a newsletter together [11]. A co-creation typology helps teachers to reflect on what type they are engaging in and communicate this to others [11]. Co-creation in medical education has predominantly been situated as a design activity *of* curricula and modules [28–31]. Little is known about the process of implementing co-creation in the clinical learning environment, as a learning and teaching activity *in* the curriculum, or about the medical student experience of co-creation in this context [11, 32]. Understanding more about how co-created learning and teaching experiences affects medical students during clinical placements might help us to better understand the value of incorporating this pedagogy into medical curricula, in the hope that this could enhance medical student belonging during clinical placements.

### Study aim

The aim of this study was to explore medical students’ experience of co-creation of learning resources within the clinical environment.

## Methods

### Ethics

We received ethical approval from the University of Edinburgh Medical Education Ethics Committee – reference number 2022/27. All participants gave written consent for data collection, data analysis and the publication of anonymised results. Participants were able to leave the study at any time without giving a reason.

### Study design

This constructivist study used interpretative phenomenology, which is a methodology devoted to understanding how people make sense of experiences within their lives [33, 34]. It is committed to detailed inquiry of each case, in its own right, in a particular context, which forms a thorough and systematic analysis [35]. The data is considered at multiple levels and stages, within an interpretative ebb and flow, in interaction with the researcher [36]. Interpretative phenomenology has particular utility when the topic in question is relatively under-researched and is related to self, identity and meaning-making [37].

Inherent in this methodology is the belief that to gain an understanding of the participants’ lived experience, rigorous interpretative work is required by the researcher. It requires the researcher to become aware of their pre-conceptions, born of their own experience, to prioritise the participants’ being-in-the world. However, there is acceptance that these pre-conceptions cannot be bracketed and hence the researcher takes a dynamic and active role [35]. We employed reflexive notes throughout the process, to continuously and cyclically re-examine the interpretation of the data to ensure the participant voice was prioritized throughout the hermeneutic cycle.

### Context

We conducted this study in the context of the primary medical degree at the University of Edinburgh in Scotland, United Kingdom. During their penultimate year, medical students undertake five-week long placements within varied clinical areas, including paediatrics. Within the tertiary paediatric hospital in Edinburgh, a monthly learning bulletin in the Acute Receiving Unit is curated by a group of paediatric trainees (residents). This resource includes key contemporaneous and condensed ‘learning points’, and signposting to further resources. It is shared via email with the entire medical paediatric team, from medical students to consultants (attendings). Paediatric trainees volunteer to be part of the five-member education team collating the learning bulletin resource. Student numbers were approximately matched to that of the paediatric trainees, by hosting two groups, asynchronously in order to provide a balance of voices from each group i.e. five specialist trainees, five students and author VIR. Further details are contained within [Additional File S1].

### Participant recruitment

Between November 2022 and January 2023, we used a purposive sampling strategy to recruit a homogenous participant group (in keeping with interpretative phenomenology methodology), all of whom were fifth-year University of Edinburgh medical students. Students were approached by author VIR during an introductory session to their paediatric clinical placement. The entire cohort, of 50 students, was informed about the study and, after having the opportunity to reflect on written information, those who wished to join the team as co-creators indicated their interest. Students were not expected to immediately indicate their interest, but were asked to contact the author via email, if they wished to participate. At the introductory session, the cohort was reassured that not participating in the study was equally acceptable, and were also informed that any co-created learning resources would be shared with the entire cohort.

### Data collection

Following their five-week placement, author VIR conducted individual semi-structured interviews regarding participants’ experiences of co-creation in the clinical learning environment. Interviews were informed by the interview guide [Additional File S2], but participants were able to talk in detail about their experiences and what was pertinent to them. Participants were encouraged to keep reflective diaries, which were informed by a set of co-created reflective questions [Additional File S3] and some participants referred to these during their interviews. Interviews were audio recorded, transcribed verbatim and data was kept confidential.

### Data analysis

**Fig. 1:**
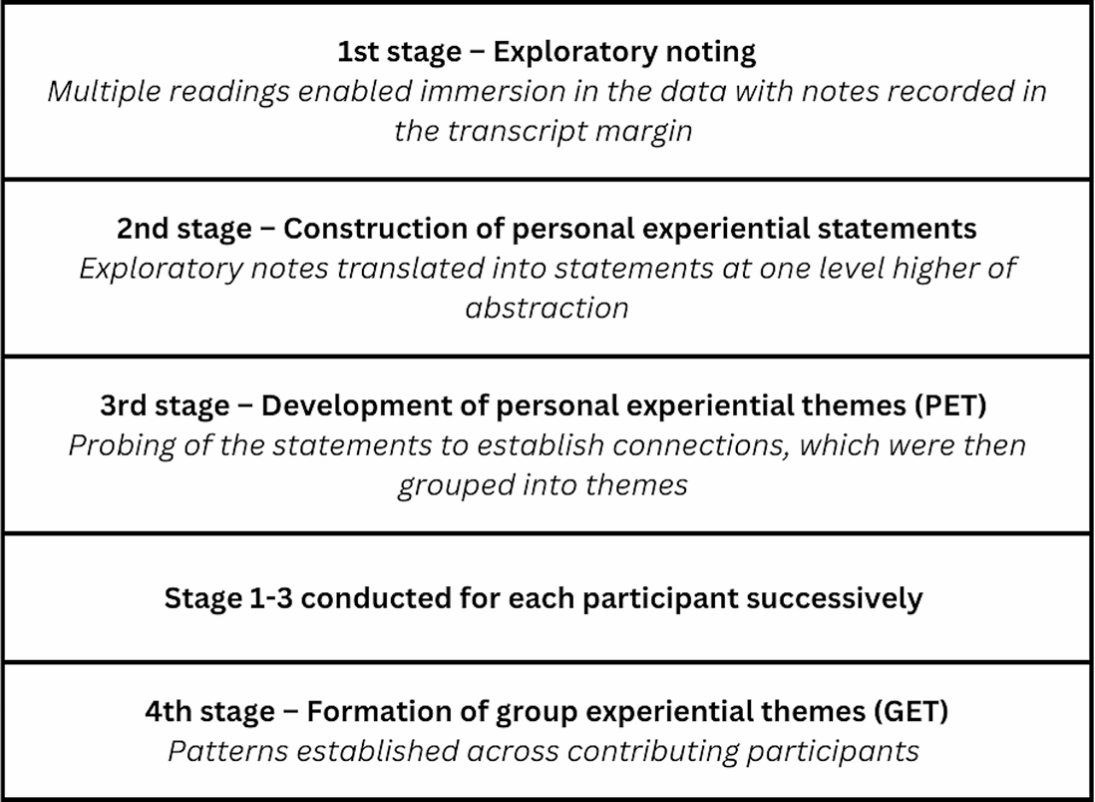
Four stage process of interpretative phenomenological analysis [35]. Table adapted from prose in Interpretative Phenomenological Analysis: Theory, Method and Research (Smith, Flowers, Larkin (2022))

We employed interpretative phenomenological analysis (IPA) of the transcribed interview data, following a four-stage process [35] [Fig. 1].

Following stage four [Fig. 1], author VIR developed the GETs into an analytic summary which was supported by quotes from each participant. VIR conducted this process with all participants, whilst VRT, SES, and SRH reviewed and audited the themes to ensure that they were grounded in the transcripts and accurately represented an interpretation of the participants’ experiences. All authors were involved in data interpretation and analysis.

### Personal and interpersonal reflexivity

Reflexive processes enabled the authors to be mindful of their worldviews and their influence on the research process. The authors hope that this reflexivity statement will enable the reader to understand how this work may align (or not) with their own beliefs, and hence the utility of the work to them. All team members are educators and researchers, and our prior research regarding identity formation [38], social relationships and integration [39, 40] are likely to have influenced themes felt to be most pertinent. Author VIR is employed as a medical education fellow by the National Health Service in Lothian (Edinburgh). A part of her job role is to support education within clinical learning environments but she does not hold responsibility for any University of Edinburgh assessment processes. VIR has the personal belief that exclusively hierarchical clinical learning environments hamper students belonging and hence learning experiences. Therefore, she believes she has a responsibility to create learning spaces for all voices to be valued [41] Additionally, she believes a competency based primary medical degree encourages enculturation of students and consequentially creates limited space for students’ own values and beliefs to be expressed. Furthermore, she believes this stifles creativity, and has a negative impact on wellbeing. Ultimately, she believes in the inherent power of personal and collective learning, which may be unlocked within a co-created learning relationship, and this has been further enhanced by becoming a co-creator of this work.

## Results

Nine students agreed to participate. The sample size is normative for IPA, where emphasis is placed on the detailed analysis of each case and not the total case number [35, 42]. IPA studies commonly have fewer than 10 participants, in order to enable researchers to undertake the detailed analysis required and uphold the idiographic focus [43, 44]. Participants were aged between 21 and 24; six identified as female, two as male and one declined to categorise their gender identity. They had no prior experience of co-creation in the clinical learning environment. Interviews were approximately half an hour in length.

**Fig. 2:**
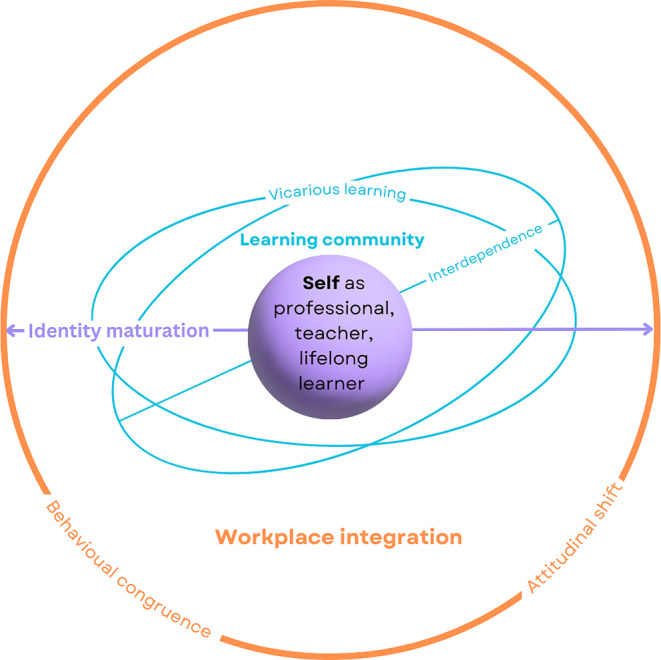
Themes and sub-themes connected across three spheres; self, co-created learning community and workplace. Interconnections between self (purple), a newly co-created learning community (blue), and the workplace (orange) are depicted within and between three spheres. Identity maturation as a professional, teacher and lifelong learner spanned all three spheres, as illustrated by the arrow moving out from the self sphere into the learning community and workplace. Interdependent learning occurred between individuals in the newly co-created learning community, represented as connecting the self sphere to the learning community sphere. Vicarious learning involved bi-directional interaction between the learning community and workplace and is hence situated between those two spheres. Behavioural congruence and attitudinal shift were found within the workplace, and are represented on the border of the workplace sphere

We identified three major themes: identity maturation; learning community; and workplace integration. The major themes, their associated sub-themes, and the relevant interconnections are depicted in Fig. 2.

### Identity maturation

Co-creation moulded how students perceived themselves in the world. Maturation was observed across a variety of their identities. Within the group experiential theme of identity maturation, we describe how the students’ views of themselves evolved as professionals; as teachers; and as lifelong learners.

#### Self as a professional

Co-creation acted as a gateway to identifying as a professional. Participants felt that they were not yet professionals, but actively sought opportunities to act in a professional role, and were grateful for the chance to do so:

*“I’ve learned a lot. Not just in terms of academic content but more as in how you work in a group as a professional…that I probably haven’t done at university yet. [To] get a feel of how you work in a team or something creative as a professional was really good.”* (Student 3)

They were motivated to become co-creators by “*trying to get somewhere now.”* (Student 5) Students felt they hadn’t experienced working authentically in a professional team and they were palpably relieved to have this opportunity:

“*To collaborate …and know that …when you put your minds together you can get things done … is reassuring.”* (Student 8)

The experience was analogous to working as a doctor, due to the development of the professional skills of negotiation, uncertainty tolerance and critical thinking:

“*Navigating through it [uncertainty], is one of the biggest challenges for new doctors. [The] co-creation project might be [a] nice segue in to this space [first-year doctor]. I will be better prepared thanks to this project.”* (Student 1)

#### Self as a teacher

Students were able to participate by becoming a teacher of the teachers, which represented a new role within the clinical learning environment. It encouraged deep engagement and was described as a “*really good way to enhance your learning even more*.” (Student 2) They also articulated that becoming a teacher of teachers felt like a courageous act, stepping away from the expected norms of a medical student:

“[This was an] *opportunity to teach against the gradient of the traditional hierarchy of seniority and step outside of the comfort zone.”* (Student 9)

They described the process of embodying the duality of being a learner, as well as a teacher:

“[To know] *what people might like to know about or what are interesting things we’re learning and what knowledge you’d like to share with other people, thinking about that whilst on placement was a good thing.”* (Student 4)

#### Self as a lifelong learner

Students’ relationships with medical knowledge were changed by the experience. They moved from seeing knowledge as an entity passed from teacher to learner – a rejection of the knower and the known. Knowledge could now be viewed as, “*communal learning, and recognising that everyone’s got knowledge that other people don’t have or might not have.” (*Student 3)

Empathy for doctors, who were now also recognised as situated on the continuum of a learning journey, was developed:

“*You’re both learners and you both are wanting to learn more about certain things. And even though one might be further along than the other, you’re both still on that continuous journey of learning.”* (Student 7)

In summary, participants were no longer solely learners. They identified with new and multiple identities of professional, teacher and lifelong learner.

### Learning community

Students experienced a new collaborative learning community where they could support, create and learn with each other. Sub-themes included interdependence and vicarious learning.

#### Interdependence

All participants experienced becoming part of a new collegiate community, which was separate to, but deeply intertwined with, the clinical learning environment. This was an opportunity to connect with others, which contrasted with participants’ usual experience of isolated working during placement. This new learning space bolstered and grounded Student 1. This was a shared experience of the group, but Student 1went on to relate it to personal wellbeing:

*“It was therapeutic. I was having ups and downs every day and the meeting actually helped me. It does encourage people to…come to others…rather than being in [their] own thoughts.”* (Student 1)

The group unified around their shared purpose of co-creating the resource and this enhanced social cohesion:

*“It feels really rewarding when you’ve all put work into something together and it comes together really nicely, that’s a nice bonding experience that you have.”* (Student 7)

This type of collaboration had previously only been experienced with trusted friends, but co-creation built trust within and beyond the co-creation group:

*“I think if you learn something interesting it’s nice to be able to share it with people. And usually… with my friend, if you learn something and you’re like, oh that was really cool and we’ll tell each other… But to… then be asked to tell more people*.” (Student 4)

Participants had an interdependent learning experience which was born from trust and generated collaborative purpose.

#### Vicarious learning

Students recognised the importance of social learning during the process of creating a new resource together. Enhanced equity of exposure to learning was possible by making visible individual clinical experiences, which were transmitted via the group:

“*I felt like I was able to…get a broader experience…that I wouldn’t have necessarily got.”* (Student 6)

Vicarious learning was a reciprocal process with new insights also taken back out into the clinical learning environment:

“*In the first couple of weeks it just made me a bit more alert and then the weeks after that, I just found it really interesting to see the themes, that we as a group, had thought would be useful, to see how they were visible. Even though it’s someone else that had observed them, once they’d started looking for them, I could also see them* [in the clinical learning environment].” (Student 3)

*“Open conversations”* (Student 5) were central to the process of vicarious learning. The conversations created a stimulus for individual reflection:

*“Thinking about those things that other people had said, those concepts, I’ve definitely reflected on those things a lot more whilst I was on placement.”* (Student 4)

Via these tools, the group came to find coherence by working through the creative process together:

*“I think if we’d known from the beginning exactly what we were doing, that would have made it easier. But then I also think that… no-one know[s] what we were doing right at the beginning and that’s part of it, isn’t it, figuring out what it is we want… how we want to be involved and what it is that we can do.”* (Student 4)

In summary, a supportive and interdependent learning community was established through multiple interrelated social and creative processes, where students worked out how they could best contribute. A sense of belonging was nurtured, as students felt they mattered, were seen and were respected within a community they were equally responsible for building.

### Workplace integration

The support found within the third space learning community, as well as the maturation of their identities, allowed the participants to experience a challenge to their existing worldview. Consequently, integration into the workplace occurred in a new way which was enacted by participants in two ways: behavioural congruence and attitudinal shift.

#### Behavioural congruence

Student’s behaviours found more alignment with their personal values. They felt more able to *“speak back and share”* (Student 6) with consultants (attendings) and paediatric trainees. There was a growing reciprocity with these staff groups as a result:

“*It’s just a bit of a snowball, I think. Once you start asking people questions and speaking to them, they’ll respond better to you.*” (Student 3)

Listening more, driven by the responsibility to co-create the learning bulletin, also contributed to this reciprocity:

*“I think having two-way conversations … when things are less clear it’s interesting to have your ear to the ground, about those kinds of conversations.”* (Student 5)

Speaking up was mediated by the social capital garnered by being part of the co-creation project:

“[*Co-creation] gave me something to talk about which can …be really helpful…, when you feel like you might not have a lot of common ground with someone.”* (Student 6)

Consultants (attendings) and paediatric trainees could not always be relied upon to support learning processes due to clinical workloads, which left students feeling lost and consequentially unproductive, which was incongruent with their values:

*“Especially at the beginning of the block, you do feel a bit purposeless. You are very much just standing in a corner waiting for someone to look out for you.”* (Student 3)

Co-creation changed this feeling of being lost and they took initiative:

“*I wondered, does it happen often [a clinical event], even though I wasn’t directly involved, [co-creating] made me feel like I was getting something out of being at placement. Whereas, before [co-creation], I would have just been like, oh I’ve sat for an hour and no one’s looked at me.”* (Student 3)

#### Attitudinal shift

Participants’ attitudes towards the clinical learning environment became more positive as result of co-creation. This mentality shift resulted in enhanced motivation, driven by increased agency. Medical student positionality and associated value was changed:

“*I think with co-creation, because it makes you, as a medical student feel valued and feel like the work that you do and the input that you have does make a difference, and people are willing to listen to you, and you do have something to offer to the team.”* (Student 7)

Students conceptualised the inclusivity of co-created learning as representative of the values of the workplace:

“*It made me feel more positively about the kind of people that I was going to be around signall[ing] to me that it wasn’t going to be a horribly hierarchical five weeks*” and, “*[it] was just a signifier that it was a more open and responsive department.”* (Student 3)

However, it was perhaps only an adjunct, reinforcing those values, when teams were already inclusive:

“*It felt like a really inclusive team, which was a lot nicer than some of my past experiences, so I think the co-creation made it feel even more inclusive and made it feel like I’m more part of the team.”* (Student 7)

Co-creation was also related to the experience of users interacting with the health service:

*“Lots of negative experiences [of patients] are borne out of feeling misunderstood or not respected, [co-creation] seems like a good way of starting to dismantle that because you are breaking down barriers and hierarchies that can feel really trapping.”* (Student 6)

In summary, the medical students experienced co-creation as a tool to support them to belong as themselves in the workplace. Across all themes, participants felt empowered by new, freeing and useful ways of being, with novel roles and symbiotic ways of connecting with others. Within this theme, participants shared how co-creation explicitly signalled to them the value they could bring, which was interpretated as them developing a sense of relational belonging.

## Discussion

This study explored participating medical students’ experiences of co-creating a learning bulletin, as an activity involving authentic professional team working. The co-created learning and teaching activity *in* the curriculum created a platform for a co-created learning community and shaped identity maturation along with new workplace behaviours and attitudes.

Within the self-sphere, students were given an invitation to embody a powerful new identity as a teacher of teachers (providing learning to those who also teach the students). Identity maturation is related to the developmental concept of self-authorship. Self-authorship is “the capacity of an individual to define a coherent internal belief system” and utilise this within relationships, decisions and actions [45]. Self-authorship is defined in three dimensions: cognitive, intrapersonal and interpersonal [46]. It applies to the co-creation experience because we observed students developing self-authorship in the cognitive dimension (adjustment of their epistemic expectations) [47], the intrapersonal dimension (different ways of being a medical student which were congruent with their personal values) and the interpersonal dimension (relationships with peers and staff were constructed and perceived differently). The self-authoring process was a vehicle for them to be empowered to belong as themselves, known as ‘relational being’ [6]. The ability of co-creation experiences to aid self-authorship has been observed in higher education students’ co-creation *of* curriculum [48], but has not previously been described in medical students engaged in a short co-creation learning and teaching activity. Becoming self-authored, via a crossroad experience such as this, is important for future healthcare professionals who will be navigating a progressively complex healthcare landscape [46].

As newly valued, increasingly self-authored beings, students were able to build social connections within the learning community and workplace spheres. Previous work on social integration divides such connections into bonds (those with shared identities) and bridges (those with different identities) [49]. Participants in our study formed bonds within the co-created learning group. As a result, they then formed bridges out into the workplace [49, 50]. This new learning network was co-regulatory and vicarious [51, 52] in nature and responds to the need to re-examine how we support student connection, which is critical to wellbeing [53]. Within this exploration, interconnectivity across the three spheres (self, learning community and workplace), showcased how social integration and belonging intersect. Hence, promotion of a post-humanism view, which considers students within a learning ecology, in contrast to prevailing transactional competitive individualism is encouraged [6]. Integration between internal and external influences has been related to professional identity formation via social cognitive theory [54]. Co-created bidirectional learning relationships and new networks are waiting to be utilised by medical educators, to encourage *all* students to integrate, and hence foster a learning ecology where everyone can ‘*be’* and participate meaningfully.

### Strengths and limitations

IPA offered a unique opportunity for an in-depth examination of *how* medical students learn whilst participating in co-creating a learning resource [55]. As with other constructivist methodologies, findings may be transferable (to some contexts), but are not generalizable (to all contexts) [56, 57]. The use of reflective diaries alongside interviews was beneficial as it encouraged participants to reflect prior to sharing their experiences. VIR was the interviewer, as well as a co-creator, which built relational trust with the participants prior to interview and gave her an ‘insider’ view. Given this, authors VRT, SES, SRH, who did not have a learning relationship with the participants, were utilised to independently verify the findings. However, the learning relationship with author VIR may have influenced what the participants chose to share about their experience, as they may have wished to express gratitude for their inclusion and VIR’s time and effort. This may have resulted in negative facets of their experience remaining undisclosed. Perceptions of existing power relations, in the clinical learning environment may have also been a mediating influence both on student experience, as well as how they felt able to share their experiences.

### Further research

It would be beneficial to understand the experience of medical students, within the cohort, who did not participate. Specifically, it would be important to understand whether the results of this study were transferable to non-participating students, who were aware of co-creation occurring in their learning environment. We would be interested to understand what factors determine students’ decisions to become co-creators, as well as directing further research towards developing our understanding of how co-creation can be inclusive for all, not just some learners. Furthermore, we are interested to understand whether there was sustained impact on identity maturation, behaviours and attitudes via self-authoring and social integration bond and bridge-building. Co-production of research with medical students is likely to be a powerful tool to develop a more nuanced understanding.

## Conclusion

Medical students in this study described experiences of identity maturation as professionals, teachers and as lifelong learners. They also co-created a new learning community, involving internal bonds and bridges out into workplace. This study lends support to the notion that co-creation may have the power to transform medical students’ experience of belonging, and may deliver meaningful learning experiences. Medical educators should consider enabling co-creation opportunities within their curricula, to promote students’ self-authorship and embodiment of helpful new professional identities.

### Electronic supplementary material

Below is the link to the electronic supplementary material.

Supplementary Material 1

Supplementary Material 2

Supplementary Material 3

## Data Availability

The datasets used and/or analysed during the current study are not publicly available, as participants did not consent to this, but are available from the corresponding author on reasonable request, if additional consent sought and gained from participants.

## Abbreviations

IPA: Interpretative phenomenological analysis
PET: Personal Experiential Themes
GET: Group Experiential Themes
