# Supplementary material for: From corners to community: exploring medical students’ sense of belonging through co-creation in clinical learning
Source: BMC Med Educ. 2024 Apr 30;24:474. doi: 10.1186/s12909-024-05413-2 (PMC11059736; doi:10.1186/s12909-024-05413-2)
Supplement: Supplementary file 1 — Supplementary Material 1 [file 12909_2024_5413_MOESM1_ESM.docx]

**Additional File 1: Learning bulletin creation within Paediatric Acute Receiving Unit**

This document outlines how the department usually creates its monthly learning bulletin. We then describe how the process was changed so that medical students and paediatric specialist trainees co-created the learning bulletin together.

**Creation of the paediatric department monthly learning bulletin**

A group of paediatric specialist trainees meet monthly to decide on the educational priorities within the acute paediatric receiving unit. The priorities are decided collaboratively between this team, and in conference with consultants (attendings) and other ward staff. However, the specialist trainees are empowered to have ownership of the learning resource. The priorities are often based on learning generated from clinical cases seen within the acute paediatric receiving unit or grounded in key national knowledge updates, e.g. new guidelines or patient safety initiatives. The learning bulletin is evidenced based. It is used to provide equity of learning across the team and it supports the whole clinical team in keeping up to date.

Following the decision-making process of what will be included, the learning bulletin team may work independently or in collaboration on parts of the bulletin. They will work asynchronously on a document housed on the platform Canva to collate written information, alongside developing relevant images and figures. One team member edits the final document, then the learning bulletin is shared via email with all members of the team located in the acute paediatric receiving unit. The team has approximately 100 members at one time, with some staff based permanently in this unit and others working within it on a rotational basis.

**Involvement of medical students to co-create the learning bulletin with the trainees**

Paediatric specialist trainees were consulted prior to the involvement of medical students in co-creating the learning bulletin. Information was shared about the project aims, and the specialist trainees agreed to medical students joining their team as co-creators.

Medical students were invited to participate on a volunteer basis as part of this study. At the outset of the project, students were prepared, firstly by meeting together. During this first meeting, we discussed how we wished to work during the project. This was not dictated by author VIR or the specialist trainees, but was decided together. We made logistical decisions such as how often we should meet and how we would communicate with each other.

Author VIR initiated an early discussion about the challenges of co-creation and students were invited to share their hopes and concerns about the process. We discussed that because this was a new process for us as a group, that how the project developed would be guided by the new co-created team. We reflected this could feel messy but all members of the co-created team agreed to embrace this together. We discussed means by which to share any concerns we had about this during the process.

At the outset, we discussed what feedback meant to the study participants, and agreed together our ways of working when delivering and receiving feedback to support learning for the whole team in this co-created learning relationship. In this first meeting, we specifically acknowledged that students giving feedback to specialist trainees and author VIR, may be challenging, given the pre-existing hierarchical learning structures. In response to this, we agreed to work within our feedback contract, which included that we would give feedback face to face so that there would be an opportunity to have dialogue around this.

Students met with author VIR on a weekly basis and communicated with her, as well as with each other and the paediatric specialist trainees, via email in between the occasions on which we met. After decisions were made about how the team would work together, students entered the process alongside the paediatric specialist trainees to decide what felt important to include in the learning bulletin. After a topic area was identified, our co-created learning community decided how to approach the generation of a new resource which would be beneficial to the entire team. This would then would be disseminated to them via email. We then followed a similar process for generating the learning bulletin. Roles were allocated dependent on individual interests and skills. Author VIR ensured the quality of the learning bulletin resource by reviewing its development throughout the process and particularly, prior to dissemination to the wider team in the acute paediatric receiving unit.

During dissemination of the learning bulletin, we shared how the medical students had participated as co-creators and this was celebrated.
