# Supplementary material for: From corners to community: exploring medical students’ sense of belonging through co-creation in clinical learning
Source: BMC Med Educ. 2024 Apr 30;24:474. doi: 10.1186/s12909-024-05413-2 (PMC11059736; doi:10.1186/s12909-024-05413-2)
Supplement: Supplementary file 2 — Supplementary Material 2 [file 12909_2024_5413_MOESM2_ESM.docx]

**Additional File 3: Co-created reflective questions**

- What actions have I taken to progress this project this week?
- What challenges have I encountered?
- What has worked?
- What are the next steps?
