# Supplementary material for: From corners to community: exploring medical students’ sense of belonging through co-creation in clinical learning
Source: BMC Med Educ. 2024 Apr 30;24:474. doi: 10.1186/s12909-024-05413-2 (PMC11059736; doi:10.1186/s12909-024-05413-2)
Supplement: Supplementary file 3 — Supplementary Material 3 [file 12909_2024_5413_MOESM3_ESM.docx]

**Additional File 2: Interview guide**

- Tell me about what being on a clinical placement feels like?
- How do you learn during placement?
- Can you describe your experience of co-creation?
  - What has it felt like?
  - Is this something you have previously experienced during medical school?
- Can you share why you decided to become part of this project?
- Has co-creation changed things for you at all?
  - Is anything different now?
  - Do you do anything differently since this co-creation experience?
  - Does anything feel different now?
- Has co-creation changed the way you think or feel about your paediatric placement?
  - What has caused the change?
  - What has brought that change about?
- Can you describe how being part of co-creation has influenced your relationships whilst on your paediatric placement?
- Has anything affected your experience of co-creation
  - What has been the challenges?
  - What has been useful?
- Is there anything that we haven’t yet discussed which you would like to share?
